# Family-Centric Applied Behavior Analysis Promotes Sustained Treatment Utilization and Attainment of Patient Goals

**DOI:** 10.7759/cureus.62377

**Published:** 2024-06-14

**Authors:** Robert P Adelson, Madalina Ciobanu, Anurag Garikipati, Natalie J Castell, Gina Barnes, Ken Tawara, Navan P Singh, Jodi Rumph, Qingqing Mao, Anshu Vaish, Ritankar Das

**Affiliations:** 1 Research and Development, Montera, Inc. DBA Forta, San Francisco, USA; 2 Engineering, Montera, Inc. DBA Forta, San Francisco, USA; 3 Clinical Team, Montera, Inc. DBA Forta, San Francisco, USA; 4 Executive Leadership, Montera, Inc. DBA Forta, San Francisco, USA

**Keywords:** treatment outcomes, telehealth, skill acquisition, parent training, autism spectrum disorder, applied behavior analysis

## Abstract

Background/objectives: Autism spectrum disorder (ASD) is a neurodevelopmental disorder characterized by social communication difficulties and restricted repetitive behaviors or interests. Applied behavior analysis (ABA) has been shown to significantly improve outcomes for individuals on the autism spectrum. However, challenges regarding access, cost, and provider shortages remain obstacles to treatment delivery. To this end, parents were trained as parent behavior technicians (pBTs), improving access to ABA, and empowering parents to provide ABA treatment in their own homes. We hypothesized that patients diagnosed with severe ASD would achieve the largest gains in overall success rates toward skill acquisition in comparison to patients diagnosed with mild or moderate ASD. Our secondary hypothesis was that patients with comprehensive treatment plans (>25-40 hours/week) would show greater gains in skill acquisition than those with focused treatment plans (less than or equal to 25 hours/week).

Methods: This longitudinal, retrospective chart review evaluated data from 243 patients aged two to 18 years who received at least three months of ABA within our pBT treatment delivery model. Patients were stratified by utilization of prescribed ABA treatment, age, ASD severity (per the Diagnostic and Statistical Manual of Mental Disorders, Fifth Edition), and treatment plan type (comprehensive vs. focused). Patient outcomes were assessed by examining success rates in acquiring skills, both overall and in specific focus areas (communication, emotional regulation, executive functioning, and social skills).

Results: Patients receiving treatment within the pBT model demonstrated significant progress in skill acquisition both overall and within specific focus areas, regardless of cohort stratification. Patients with severe ASD showed greater overall skill acquisition gains than those with mild or moderate ASD. In addition, patients with comprehensive treatment plans showed significantly greater gains than those with focused treatment plans.

Conclusion: The pBT model achieved both sustained levels of high treatment utilization and progress toward patient goals. Patients showed significant gains in success rates of skill acquisition both overall and in specific focus areas, regardless of their level of treatment utilization. This study reveals that our pBT model of ABA treatment delivery leads to consistent improvements in communication, emotional regulation, executive functioning, and social skills across patients on the autism spectrum, particularly for those with more severe symptoms and those following comprehensive treatment plans.

## Introduction

Autism spectrum disorder (ASD) is a highly heterogeneous neurodevelopmental condition characterized by social impairments and restricted repetitive behaviors or interests (RRBIs) [1]. In the United States (US), the prevalence of ASD among children eight years of age was estimated in 2020 to be one in 36, a number that has experienced continuous growth over approximately 20 years [2,3]. The impact of ASD on interpersonal relationships and daily living skills is lifelong, and although difficult to quantify, financial costs for ASD from care-related expenses and productivity losses are estimated to reach over $400B by 2025 [4]. Thus, a broad implementation of evidence-based interventions to mitigate the impact of ASD is critical [5-7].

The gold standard of ASD treatment is applied behavior analysis (ABA), which leverages positive reinforcement to teach constructive and necessary skills across a variety of domains, including socialization, communication, emotional regulation, and executive function [8-14]. ABA principles involve reducing or encouraging the uptake of a target behavior by altering the environment or scenario that is driving a particular behavior in order to systematically encourage a different desired response that is generalizable within diverse situations [8-14]. ABA treatment plans are highly individualized and treatment response is highly variable [15]. Treatment plans are developed by a board-certified behavior analyst (BCBA) in accordance with guidelines from the Behavior Analyst Certification Board (BACB) [12]. Treatment plans include goals to learn or refine skills that encompass domains often lacking in individuals on the autism spectrum, including communication, emotional regulation, executive functioning, and social skills [16-21]. Acquisition of these skills is vital for therapeutic success [17,19-23]. Depending on a patient’s weakness in any of these skills, a dosage of ABA treatment will be prescribed following in-depth evaluation by a BCBA. The number of weekly hours of treatment prescribed to a patient is typically 10-40 hours [12]. After receiving an individualized treatment plan, ABA is administered by a behavior technician (BT) under the clinical supervision of a BCBA.

Because of the significant time commitment required from qualified professionals (i.e., BCBAs and BTs) to implement ABA treatment and growing ASD prevalence, there is an overwhelming demand for ABA services, which has resulted in families in need of ABA services struggling to find providers and/or facing long delays on treatment waitlists [24-27]. Additional factors also impact families’ ability to seek care, including cost, geographic limitations, and scheduling constraints [28-30]. Collectively, these barriers mean that many children on the autism spectrum are unable to receive care or receive treatment far below the recommended level [31-33].

In order to overcome these barriers to accessing care, Montera, Inc. DBA Forta (hereinafter, Forta), an ABA service provider, developed a model of ABA that trains parents and/or caregivers as parent BTs (pBTs) [34]. In the family-centric Forta model of ABA treatment, pBTs are able to deliver care directly to their children in everyday, naturalistic settings, with ongoing supervision, guidance, and support from a BCBA and/or another professional BT. In addition to facilitating ABA access, our pBT treatment model utilizes technology to inform data-driven decisions and streamline treatment delivery.

ABA treatment plans are flexibly designed to correspond with the highly variable manifestation of ASD. However, the wide range both in ASD severity and in treatment plan design means that the outcomes of treatment are similarly varied, and factors that impact response to treatment remain poorly understood but may include patient characteristics such as cognitive functioning, ASD severity level, female sex at birth, native language, comorbid psychological conditions, and baseline verbal skills, which can affect the prescribed treatment intensity, and sociodemographic characteristics [16,22,35-42].

In this retrospective cohort analysis, we utilized our extensive database consisting of data resulting from pBT treatment implementation, including treatment utilization and progress in skill acquisition goals, to understand how patient characteristics affect ABA treatment outcomes. We hypothesized that implementation of the pBT ABA model would lead to significantly improved outcomes, as indicated by increased success rates in acquiring new skills, for patients having severe ASD when compared to those with moderate or mild ASD. In addition, we explored a secondary hypothesis that patients with comprehensive treatment plans would show greater improvements in success rates when compared to patients with focused treatment plans.

## Materials and methods

Study design

The retrospective patient treatment data presented in this work were collected as a part of the standard documentation practices used by Forta during pBT-administered ABA treatment. All data were de-identified before analysis to ensure compliance with the Health Insurance Portability and Accountability Act (HIPAA) and in accordance with the 1964 Helsinki Declaration, as revised in 2013. This study protocol was approved with an exemption determination per 21CFR56.104 and 45CFR46.104(b)(4) and received a waiver of informed consent by the Ethics Committee of Pearl Institutional Review Board (IRB) (Indianapolis, IN, USA; protocol number 22-Mont-102).

To evaluate the overall effectiveness and the importance of patient characteristics, such as age, ASD severity, treatment plan type, and treatment utilization in the context of our pBT model of ABA treatment delivery, a retrospective cohort analysis was performed to compare the mean success rates of patients over the course of six months of pBT-delivered treatment. This period substantially aligns with the execution of a single treatment plan, as BCBAs reassess each patient to adjust their treatment plan and set new patient goals approximately every six months. Treatment utilization was defined as the percentage of treatment time completed per week (in hours), relative to the number of weekly treatment hours prescribed by a BCBA for that patient. To assess the primary hypothesis that more severe ASD symptoms would be associated with larger gains in skill acquisition, patients were stratified by ASD severity level according to the Diagnostic and Statistical Manual of Mental Disorders, Fifth Edition (DSM-5) into mild, moderate, and severe cohorts, corresponding to Levels 1, 2, and 3, respectively, of severity for both social communication impairments and RRBIs. In addition, for our secondary hypothesis, that patients with comprehensive treatment plans achieve greater progress in skill acquisition goals compared to those with focused treatment plans, patients were stratified according to their weekly prescribed treatment dosage. For all patients in our study cohort, progress was assessed by examining the success rates during treatment sessions over time, both overall and within individual focus areas.

Participant selection

**Figure 1:**
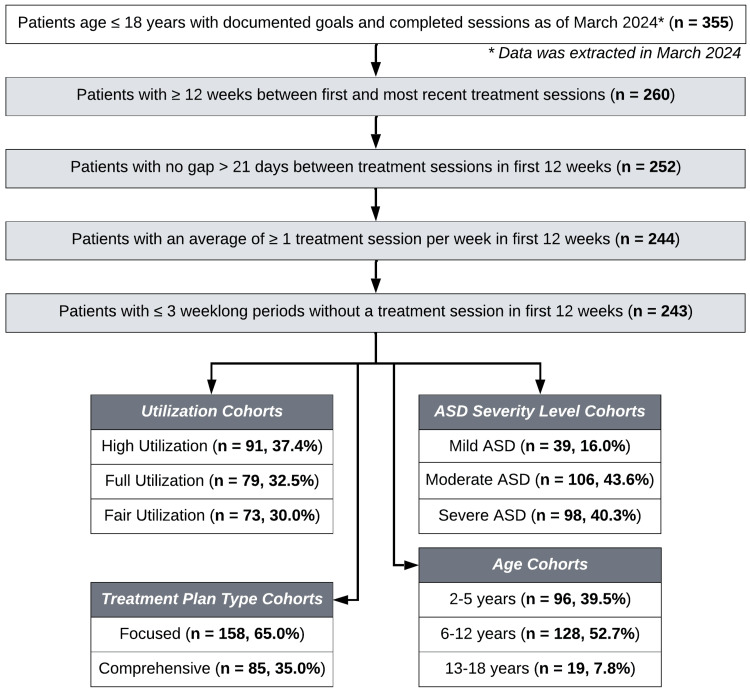
Attrition chart for the selection and inclusion of patients in the study cohort. ASD = autism spectrum disorder

Patients receiving ABA treatment with Forta using our pBT model with available data between October 2022 and March 2024 (when data were extracted) were eligible for inclusion. Complete documentation of goal progress for at least three consecutive months of treatment was required; the process of identifying patients who met the study requirements is shown in Figure 1. A total of 243 patients had at least three months of complete treatment records and were included in this study.

Demographic and clinical data used for patient stratification in this analysis were obtained from treatment intake forms completed by the pBT and from the BCBA-developed treatment plans (Table 1). Patients were classified into cohorts based on the utilization of the prescribed treatment, age, ASD severity level (per DSM-5), and the intensity of the prescribed treatment dosage (treatment plan type).

**Table 1:** Patient demographics for the patient study cohort. ADHD = attention deficit hyperactivity disorder; ASD = autism spectrum disorder; DSM-5 = Diagnostic and Statistical Manual of Mental Disorders, Fifth Edition; GDD = global developmental delay; pBT = parent behavior technician; SEM = standard error of the mean

Demographic category	Category value	Analysis cohort (n = 243)	Overall mean (SEM)
Patient age at the first treatment session (years)	2-5	96 (39.5%)	7.0 (0.2)
-	6-12	128 (52.7%)	-
-	13-18	19 (7.8%)	-
Patient age at diagnosis (years)	0-4	148 (60.9%)	4.6 (0.2)
-	5-8	72 (29.6%)	-
-	9-17	23 (9.5%)	-
Sex assigned at birth	Male	184 (75.7%)	n/a
-	Female	59 (24.3%)	-
Payor type	Private	107 (44.0%)	n/a
-	Public	136 (56.0%)	-
ASD severity level (DSM-5)	Mild	39 (16.0%)	n/a
-	Moderate	106 (43.6%)	-
-	Severe	98 (40.3%)	-
Treatment plan type	Focused	158 (65.0%)	n/a
-	Comprehensive	85 (35.0%)	-
Utilization (%)	Fair (<80)	73 (30.0%)	84.6 (1.4)
-	Full (80-95)	79 (32.5%)	-
-	High (>95)	91 (37.4%)	-
Schooling type	Home	57 (23.5%)	n/a
-	Regular	125 (51.4%)	-
-	Special education	30 (12.3%)	-
-	None	30 (12.3%)	-
-	Unknown	1 (0.4%)	-
Prior/concurrent therapy	Prior ABA therapy	67 (27.6%)	n/a
-	Speech therapy	93 (38.3%)	-
-	Occupational therapy	71 (29.2%)	-
-	Physical therapy	14 (5.8%)	-
-	Other	25 (10.3%)	-
Comorbidities	ADHD	39 (16.0%)	n/a
-	Language disorders	21 (8.6%)	-
-	Anxiety	11 (4.5%)	-
-	Sensory processing	4 (1.6%)	-
-	GDD	7 (2.9%)	-
-	Other	12 (4.9%)	-
pBT race/ethnicity	White	97 (39.9%)	n/a
-	Black or African-American	43 (17.7%)	-
-	Hispanic or Latino	36 (14.8%)	-
-	Asian	12 (4.9%)	-
-	American Indian or Alaska Native	4 (1.6%)	-
-	Native Hawaiian or Other Pacific Islander	2 (0.8%)	-
-	Two or more races	4 (1.6%)	-
-	Declined to answer	45 (18.5%)	-

Intervention delivery

Forta’s treatment model uses a family-centric framework that employs technology to streamline treatment. Parents become qualified to act as BTs and provide formal delivery of ABA treatment to their children after a rigorous standard-of-care training program consisting of instruction on validated ABA methodologies and adhering to the BACB standard of care for ABA delivery. Training occurs over 50 hours via an online platform that includes both synchronous and asynchronous instructional modules. After training, pBTs are required to pass an Initial Competency Assessment to demonstrate the skills and knowledge required to deliver ABA treatment. The first attempt pass rate of this assessment is 86% for Forta pBTs. BCBAs provide ongoing, close supervision and instructional feedback of all pBTs via telehealth, which meets or exceeds 5% of treatment time, as established by the BACB guidelines. In Forta’s family-centric model of ABA treatment delivery, in addition to BCBA support, another professional BT may provide treatment support to the pBT via telehealth. During ABA delivery, pBTs and BCBAs receive access to tools to streamline their workflows. This includes a proprietary web-based, HIPAA-compliant application ("app") that allows a pBT to review their child's treatment plan, log treatment hours, and track progress toward attaining individual patient goals; BCBAs additionally have access to software that uses individual patient information to make data-driven suggestions regarding which treatment plan (focused vs. comprehensive) is likely optimal for each patient [34,43].

As in conventional ABA (i.e., non-pBT delivered ABA), treatment plans were highly individualized to meet patient needs. Treatment plans were created by BCBAs within our proprietary app and were developed in accordance with the BACB guidelines. At intake, BCBAs evaluated metrics from validated assessments, such as the Vineland Adaptive Behavior Scales, Third Edition (Vineland-3), to create treatment plans. Vineland-3 is a validated assessment tool used in the context of ASD to measure an individual’s adaptive behavior and treatment progress [34,44,45]. Treatment plans included individualized strategies and patient goals, as well as a treatment dosage recommendation (i.e., number of hours of prescribed weekly treatment). The pBT Forta model employs a rigorous requirement for BCBAs to facilitate scheduling supervision sessions with pBTs at least weekly; in addition, BCBAs and clinical directors meet regularly to ensure that all patients are achieving expected progress toward skill acquisition.

Outcome measurements

Utilization rates were obtained from the treatment information recorded in the app by pBTs. Utilization was calculated for each week of treatment to track the percentage of prescribed weekly ABA being completed over the course of treatment. In some instances, utilization rates >100% occurred when patients received more hours of weekly treatment than was prescribed. ASD severity level (i.e., mild, moderate, and severe) for each patient was collected from routine patient intake forms and the patient’s plan type (i.e., focused or comprehensive) was confirmed within each patient’s treatment plan by the study team.

Baseline and longitudinal success rates toward skill acquisition within four focus areas (communication, emotional regulation, executive functioning, and social skills) were evaluated for each individual patient (n = 243), with data collected during routine and ongoing treatment. These focus areas correspond with specific domains or subdomains of the Vineland-3, with communication corresponding to the Communication domain, emotional regulation to the Coping Skills subdomain (within the Socializing domain), executive functioning to the Daily Living Skills domain, and social skills to the Interpersonal Relationships subdomain and Play and Leisure subdomain (both within the Socializing domain). Patient progress within these focus areas was assessed by tracking success rates during treatment delivery as recorded in the app by pBTs during each treatment session. The success rate was calculated for each skill acquisition goal within each session for each patient, and these values were subsequently averaged between all goals within a focus area for a particular patient in a particular week, and those averaged values were themselves averaged across all patients during that particular week of treatment. The success rate in acquiring skills served as an indicator of the patients’ level of achievement toward specific goals established within their treatment plans. During a treatment session, a patient was prompted a specific number of times to attempt a behavior. The success rate represents the net percentage of successful attempts to perform a behavior out of all attempts for that specific behavior during a treatment session. These are reported as a percentage value ranging from 0% (no successful attempts) to 100% (all attempts were successful). Typically, success rates reaching 80-100% are considered skill mastery in ABA treatment implementation [46]. New goals are initiated when this success rate threshold is reached.

Statistical analysis

We employed descriptive statistics - mean and standard error of the mean (SEM) - to examine the distribution of participants across various cohorts based on utilization, age, ASD severity level, and treatment plan type. Pairwise comparisons between cohorts were performed using two-tailed Student’s t-tests with a significance level of *p *< 0.05 indicating statistical significance. Mean and SEM were also calculated to summarize patient success toward attaining treatment goals, in terms of the mean net change (ΔMean) in goal success rate from baseline to week 26 across different cohorts, both overall and within each focus area. Pairwise comparisons between cohorts were similarly performed using two-tailed Student’s t-tests with a significance level of *p* < 0.05 indicating statistical significance. To further investigate the change in success rates over time, both overall and within each focus area, Pearson's correlation coefficient (r) was calculated, and a Wald test with a t-distribution of the test statistic was conducted to test the null hypothesis that the slope of the linear regression was zero. Significant results (*p* < 0.05) were interpreted as evidence to reject the null hypothesis and suggested a significant non-zero linear relationship between the variables. For each *t*-test, the *t*-statistic and *p*-value are reported.

## Results

After patient selection as described previously, 243 patients had data available for analysis and were included in the study cohort (Figure 1). The mean age of the study cohort was 7.0 years (SEM = 0.2 years), and a majority of patients were assigned male sex at birth (n = 184, 75.7%), which corresponds with the higher prevalence of ASD diagnosed in male children [47,48]. Patient age distributions at diagnosis and at their first treatment session are shown in Table 1, with patients stratified into age cohorts corresponding to those used in previous ABA research (i.e., two to five, six to 12, and 13-18 years) [49]. A larger number of patients used public insurance (n = 136, 56.0%) compared to private insurance (n = 107, 44.0%), a significantly greater proportion than in the 2022 US general population (65.6% private, 36.1% public; Chi-square test, χ2 = 44.5, *p *< 0.001) [50]. Patients were racially and ethnically diverse, with White, Black, Hispanic/Latino, Asian, and two or more races represented in our cohort, as indicated by the parent/pBTs racial or ethnic identity (Table 1). Comorbidities represented in the full study cohort included attention deficit hyperactivity disorder (ADHD), language disorders, anxiety, sensory processing disorders, global developmental delay (GDD), and non-categorized (others).

Utilization

**Figure 2:**
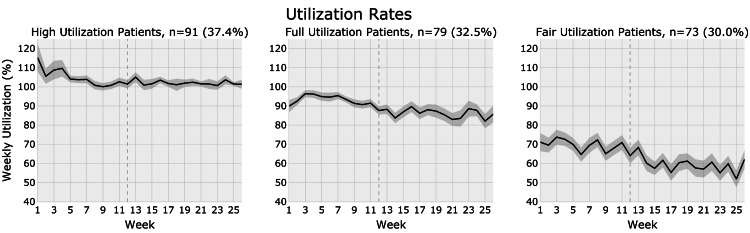
Utilization cohorts displaying the variation in weekly utilization for the study duration. The black line represents the mean utilization for a week, while the shaded region represents the standard error of the mean (SEM) for that week.

Patients were stratified based on utilization rate into high utilization (≥95% mean utilization; n = 91, 37.4%), full utilization (80-95% utilization; n = 79, 32.5%), and fair utilization (<80% utilization; n = 73, 30.0%) cohorts. There were no significant differences in mean patient age and mean prescribed treatment dosage across utilization cohorts, and each utilization cohort contained patients of all ASD severity levels (Table 2). The high, full, and fair utilization cohorts had a mean utilization of 102.3%, 87.7%, and 58.9%, respectively. The longitudinal variation in mean utilization over time for each cohort is shown in Figure 2a. The utilization rate remained relatively stable within each utilization category over time within the six-month period investigated; the mean utilization across the study population was 84.6%. In the high utilization cohort, weekly utilization was greatest at baseline (>100%) and later stabilized around 100% starting at approximately week 5. The full and fair utilization cohorts demonstrated a gradual decline in utilization over time, which was most pronounced for the fair utilization cohort. The mean for the full utilization cohort did not drop below 80% utilization at any point during the six-month period. Patients completed an average of 3.0 (SEM = 0.1) sessions/week. Table 3 details how many patients in each utilization cohort received treatment in a given week. For example, in the high utilization cohort, 91 patients (100.0%) had 12 weeks of data, 88 patients (96.7%) had 13 weeks of data, 77 patients (84.6%) had 18 weeks of data, and 69 patients (75.8%) had all 26 weeks of data. From the 77 patients in the high utilization cohort that had the necessary data through week 18, 76 patients (98.7%) received treatment during week 18, and one patient did not receive treatment during week 18 (and thus their data were not used for the utilization and success rates reported for week 18). For all patients, week 1 represents the baseline corresponding to treatment initiation using the pBT delivery model.

**Table 2:** Summary table indicating the characteristics of each utilization cohort, ASD severity level cohort, treatment plan type cohort, and age cohort. Characteristics include ASD severity level, mean age, mean utilization, mean prescribed treatment hours, and mean number of goals. Standard error of the mean (SEM) is shown for each mean value, along with a t-statistic and p-value indicating the significance of the difference between means between cohorts (calculated using a two-tailed t-test for independent means). ASD = autism spectrum disorder; Compr. = comprehensive; DSM-5 = Diagnostic and Statistical Manual of Mental Disorders, Fifth Edition; Mod. = moderate

-	-	Utilization cohort	-	-	ASD severity level cohort (DSM-5)	-	-	Treatment plan type cohort	-	Age at first treatment session (years) cohort	-	-
-	-	High	Full	Fair	Mild	Mod.	Severe	Focused	Compr.	2-5	6-12	13-18
-	-	n = 91 (37.4%)	n = 79 (32.5%)	n = 73 (30.0%)	n = 39 (16.0%)	n = 106 (43.6%)	n = 98 (40.3%)	n = 158 (65.0%)	n = 85 (35.0%)	n = 96 (39.5%)	n = 128 (52.7%)	n = 19 (7.8%)
ASD severity level (DSM-5)	Mild	19 (7.8%)	16 (6.5%)	4 (1.6%)	39 (16.0%)	-	-	32 (13.2%)	7 (2.9%)	14 (5.8%)	23 (9.5%)	2 (0.8%)
-	Mod.	36 (14.8%)	30 (12.3%)	40 (16.4%)	-	106 (43.6%)	-	72 (29.6%)	34 (14.0%)	34 (14.0%)	59 (24.3%)	13 (5.3%)
-	Severe	36 (14.8%)	33 (13.6%)	29 (11.9%)	-	-	98 (40.3%)	54 (22.2%)	44 (18.1%)	48 (19.8%)	46 (18.9%)	4 (1.6%)
Age (years)	Mean ± SEM	7.5 ± 0.4	6.9 + 0.3	6.5 ± 0.4	7.3 ± 0.5	7.6 ± 0.4	6.2 ± 0.3	7.8 ± 0.3	5.6 ± 0.3	4.0 ± 0.1	8.2 ± 0.2	14.7 ± 0.4
-	Cohort Differences	High vs. Full: *t*=1.7, *p*=0.243	Full vs. Fair: *t*=0.8, *p*=0.421	High vs. Fair: *t*=1.7, *p*=0.083	Mild vs. Mod: *t*=0.4, *p*=0.680	Mod. vs. Severe: *t*=2.8, *p*=0.006	Mild vs. Severe: *t*=1.9, *p*=0.056	Focused vs. Compr.: *t*=4.7, *p*<0.001	Focused vs. Compr.: *t*=4.7, *p*<0.001	2-5 vs. 6-12: *t*=17.0, *p*<0.001	6-12 vs. 13-18: *t*=12.0, *p*<0.001	2-5 vs. 13-18: *t*=37.5, *p*<0.001
Utilization, %	Mean ± SEM	102.3 ± 0.9	87.7 ± 0.5	58.9 ± 2.1	93.3 ± 1.9	83.0 ± 2.0	82.8 ± 2.4	85.3 ± 1.7	83.0 ± 2.2	82.0 ± 2.0	86.1 ± 2.0	86.8 ± 4.4
-	Cohort Differences	High vs. Full: *t*=13.6, *p*<0.001	Full vs. Fair: *t*=13.8, *p*<0.001	High vs. Fair: *t*=20.4, *p*<0.001	Mild vs. Mod.: *t*=3.0, *p*=0.004	Mod. vs. Severe: *t*=0.1, *p*=0.949	Mild vs. Severe: *t*=2.6 *p*=0.010	Focused vs. Compr.: *t*=0.8, *p*=0.416	Focused vs. Compr.: *t*=0.8, *p*=0.416	2-5 vs. 6-12: *t*=1.4, *p*=0.157	6-12 vs. 13-18: *t*=0.1, *p*=0.898	2-5 vs. 13-18: *t*=1.0, *p*=0.330
Prescribed hours	Mean ± SEM	23.2 ± 0.7	25.1 ± 0.7	24.6 ± 0.7	20.9 ± 1.0	23.7 ± 0.6	26.2 ± 0.6	20.4 ± 0.3	31.4 ± 0.3	27.0 ± 0.6	23.0 ± 0.5	18.8 ± 1.5
-	Cohort Differences	High vs. Full: *t*=1.9, *p*=0.058	Full vs. Fair: *t*=0.5, *p*=0.615	High vs. Fair: *t*=1.4, *p*=0.164	Mild vs. Mod.: *t*=2.4, *p*=0.017	Mod vs. Severe: *t*=2.9, *p*=0.004	Mild vs. Severe: *t*=4.6, *p*<0.001	Focused vs. Compr.: *t*=23.7, *p*<0.001	Focused vs. Compr.: *t*=23.7, *p*<0.001	2-5 vs. 6-12: *t*=5.1, *p*<0.001	6-12 vs. 13-18: *t*=3.0, *p*=0.004	2-5 vs. 13-18: *t*=5.5, *p*<0.001
No. of goals	Mean ± SEM	17.5 ± 0.6	17.4 ± 0.6	15.2 ± 0.5	16.6 ± 0.7	16.7 ± 0.5	16.9 ± 0.6	15.9 ± 0.4	18.4 ± 0.6	17.3 ± 0.5	17.1 ± 0.4	12.3 ± 0.8
-	Cohort Differences	High vs. Full: *t*=0.1, *p*=0.907	Full vs. Fair: *t*=2.8, *p*=0.006	High vs. Fair: *t*=2.9, *p*=0.005	Mild vs. Mod.: *t*=3.0, *p*=0.004	Mod. vs. Severe: *t*=0.3, *p*=0.797	Mild vs. Severe: *t*=1.1, *p*=0.261	Focused vs. Compr.: *t*=3.6, *p*<0.001	Focused vs. Compr.: *t*=3.6, *p*<0.001	2-5 vs. 6-12: *t*=0.3, *p*=0.752	6-12 vs. 13-18: *t*=4.4, *p*<0.001	2-5 vs. 13-18: *t*=4.2, *p*<0.001

**Table 3:** Number of patients contributing to the reported data on a weekly basis for the utilization cohorts.

High utilization, n=91 (37.4%)	-	-	-	-	-	-	-	-	-	-	-	-	-	-	-	-	-	-	-	-	-	-	-	-	-	-
Week number	1	2	3	4	5	6	7	8	9	10	11	12	13	14	15	16	17	18	19	20	21	22	23	24	25	26
Patients remaining	91	91	91	91	91	91	91	91	91	91	91	91	89	85	84	84	84	77	76	75	75	75	73	73	71	69
Patients on this week	91	86	87	88	86	87	86	86	84	89	89	87	85	81	82	83	84	76	73	75	75	73	69	69	67	67
Patients off this week	0	5	4	3	5	4	5	5	7	2	2	4	4	4	2	1	0	1	3	0	0	2	4	4	4	2
% of Patients off	0.0	5.5	4.4	3.3	5.5	4.4	5.5	5.5	7.7	2.2	2.2	4.4	4.5	4.7	2.4	1.2	0.0	1.3	3.9	0.0	0.0	2.7	5.5	5.5	5.6	2.9
Full utilization, n=79 (32.5%)	-	-	-	-	-	-	-	-	-	-	-	-	-	-	-	-	-	-	-	-	-	-	-	-	-	-
Week number	1	2	3	4	5	6	7	8	9	10	11	12	13	14	15	16	17	18	19	20	21	22	23	24	25	26
Patients remaining	79	79	79	79	79	79	79	79	79	79	79	79	77	75	72	72	71	61	57	53	51	50	46	44	43	42
Patients on this week	79	78	75	73	74	73	72	75	75	76	74	76	76	72	70	69	66	56	53	49	49	48	42	43	42	38
Patients off this week	0	1	4	6	5	6	7	4	4	3	5	3	1	3	2	3	5	5	4	4	2	2	4	1	1	4
% of Patients off	0.0	1.3	5.1	7.6	6.3	7.6	8.9	5.1	5.1	3.8	6.3	3.8	1.3	4.0	2.8	4.2	7.0	8.2	7.0	7.5	3.9	4.0	8.7	2.3	2.3	9.5
Fair utilization, n=73 (30.0%)	-	-	-	-	-	-	-	-	-	-	-	-	-	-	-	-	-	-	-	-	-	-	-	-	-	-
Week number	1	2	3	4	5	6	7	8	9	10	11	12	13	14	15	16	17	18	19	20	21	22	23	24	25	26
Patients remaining	73	73	73	73	73	73	73	73	73	73	73	73	71	67	65	62	61	60	56	55	55	53	51	51	47	42
Patients on this week	73	65	63	67	61	61	64	64	65	62	60	59	60	59	55	51	53	54	48	48	47	46	44	47	38	34
Patients off this week	0	8	10	6	12	12	9	9	8	11	13	14	11	8	10	11	8	6	8	7	8	7	7	4	9	8
% of Patients off	0.0	11.0	13.7	8.2	16.4	16.4	12.3	12.3	11.0	15.1	17.8	19.2	15.5	11.9	15.4	17.7	13.1	10.0	14.3	12.7	14.5	13.2	13.7	7.8	19.1	19.0

**Figure 3:**
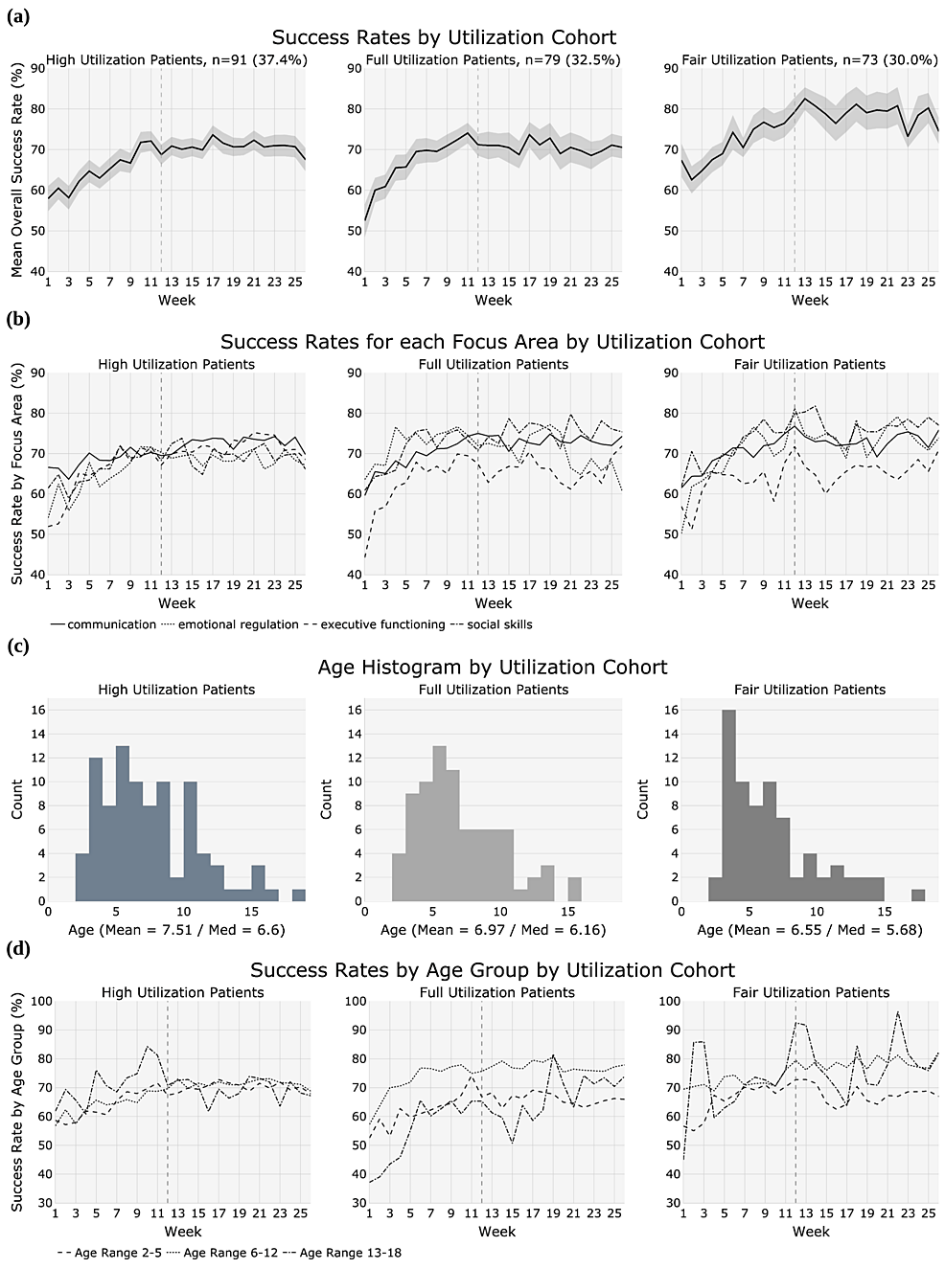
Utilization cohorts displaying (a) the mean success rate in overall skill acquisition; (b) success rate in skill acquisition in specific focus areas; (c) age histograms; and (d) success rate in skill acquisition for three different age cohorts (two to five, six to 12, and 13-18 years old).

The mean success rates over time for each utilization cohort are presented in Figure 3. The full utilization cohort generally demonstrated higher success rate net gains over the treatment period, calculated as the change in mean (ΔMean) in the success rate of skill acquisition (Table 4), than the high and fair utilization cohorts. At baseline, the high utilization cohort had its lowest success rate in executive functioning, closely followed by emotional regulation, with the highest baseline success rate in communication. The full utilization cohort similarly had its lowest baseline success rate in executive functioning, but showed the highest baseline success rate for emotional regulation. Within the fair utilization cohort, emotional regulation showed the lowest and social skills the highest baseline success rates. Executive functioning success rate lagged behind other focus areas in the full and fair utilization cohorts throughout the treatment period, but not in the high utilization cohort. Although the high utilization cohort had the smallest overall ΔMean success rate (Table 4), this cohort showed the most consistency in gains across all focus areas. Similarly, the high utilization cohort showed the most consistency in treatment response across the three age cohorts (Figure 3d); the age distribution between the utilization groups was consistent (Figure 3c) with no significant difference in utilization between age cohorts (Table 2). Furthermore, the teenage (13-18 years) cohort had significantly higher improvements overall (ΔMean = 21.7%) and in the focus areas of emotional regulation (ΔMean =31.4%), executive functioning (ΔMean = 46.1%), and social skills (ΔMean = 13.6%) compared to the younger cohorts (Table 4). However, improvements in communication skills in the teenage cohort were much lower (ΔMean = 1.9%) than in the younger cohorts (two to five years, ΔMean = 10.8%; six to 12 years, ΔMean = 9.8%).

**Table 4:** Summary table indicating the mean net change (ΔMean) in success rate in skill acquisition (from baseline to week 26) overall and across each focus area for the different utilization cohorts, ASD severity level cohorts, treatment plan type cohorts, and age cohorts. Standard error of the mean (SEM) is shown for each ΔMean success rate, along with a t-statistic and p-value indicating the significance of the difference between means (calculated using a two-tailed Student’s t-test for independent means). ASD = autism spectrum disorder; COMM = communication; Compr. = comprehensive; EF = executive functioning; ER = emotional regulation; Mod. = moderate; SOC = social skills

-	-	Utilization cohort	-	-	ASD severity level cohort (DSM-5)	-	-	Treatment plan type cohort	-	Age at first treatment session (years) cohort	-	-
-	-	High	Full	Fair	Mild	Mod.	Severe	Focused	Compr.	2-5	6-12	13-18
-	-	n=91 (37.4%)	n=79 (32.5%)	n=73 (30.0%)	n=39 (16.0%)	n=106 (43.6%)	n=98 (40.3%)	n=158 (65.0%)	n=85 (35.0%)	n=96 (39.5%)	n=128 (52.7%)	n=19 (7.8%)
COMM	ΔMean ± SEM	3.2 ± 0.4	14.7 ± 0.5	14.3 ± 0.7	7.9 ± 0.8	6.9 ± 0.4	12.7 ± 0.4	6.5 ± 0.2	13.6 ± 0.5	10.8 ± 0.4	9.8 ± 0.3	1.9 ± 3.6
-	Cohort Differences	High vs. Full: *t*=18.2, *p*<0.001	Full vs. Fair: *t*=0.5, *p*=0.639	High vs. Fair: *t*=14.4, *p*<0.001	Mild vs. Mod.: *t*=1.2, *p*=0.224	Mod. vs. Severe: *t*=10.2, *p*<0.001	Mild vs. Severe: *t*=5.9, *p*<0.001	Focused vs. Compr.: *t*=15.5, *p*<0.001	Focused vs. Compr.: *t*=15.5, *p*<0.001	2-5 vs. 6-12: *t*=2.0, *p*=0.042	6-12 vs. 13-18: *t*=5.0, *p*<0.001	2-5 vs. 13-18: *t*=4.9, *p*<0.001
ER	ΔMean ± SEM	13.0 ± 0.7	–2.8 ± 1.1	23.6 ± 1.4	14.0 ± 1.5	14.7 ± 0.9	5.8 ± 0.9	3.7 ± 0.5	23.3 ± 0.9	11.5 ± 0.8	10.0 ± 0.7	31.4 ± 6.6
-	Cohort Differences	High vs. Full: *t*=12.4, *p*<0.001	Full vs. Fair: *t*=15.0, *p*<0.001	High vs. Fair: *t*=7.2, *p*<0.001	Mild vs. Mod.: *t*=0.4, *p*=0.688	Mod. vs. Severe: *t*=7.0, *p*<0.001	Mild vs. Severe: *t*=4.8, *p*<0.001	Focused vs. Compr.: *t*=20.7, *p*<0.001	Focused vs. Compr.: *t*=20.7, *p*<0.001	2-5 vs. 6-12: *t*=1.4, *p*=0.160	6-12 vs. 13-18: *t*=6.9, *p*<0.001	2-5 vs. 13-18: *t*=5.9, *p*<0.001
EF	ΔMean ± SEM	16.6 ± 0.6	27.7 ± 1.0	14.0 ± 1.2	2.1 ± 1.2	16.0 ± 0.8	23.9 ± 0.7	17.3 ± 0.5	20.6 ± 0.8	18.2 ± 0.6	17.9 ± 0.6	46.1 ± 4.7
-	Cohort Differences	High vs. Full: *t*=9.8, *p*<0.001	Full vs. Fair: *t*=8.8, *p*<0.001	High vs. Fair: *t*=2.1, *p*=0.042	Mild vs. Mod.: *t*=9.2, *p*<0.001	Mod. vs. Severe: *t*=7.4, *p*<0.001	Mild vs. Severe: *t*=16.2, *p*<0.001	Focused vs. Compr.: *t*=3.7, *p*<0.001	Focused vs. Compr.: *t*=3.7, *p*<0.001	2-5 vs. 6-12: *t*=0.3, *p*=0.729	6-12 vs. 13-18: *t*=11.9, *p*<0.001	2-5 vs. 13-18: *t*=11.3, *p*<0.001
SOC	ΔMean ± SEM	4.7 ± 0.5	14.5 ± 0.7	15.3 ± 0.9	11.6 ± 0.9	6.5 ± 0.5	11.3 ± 0.7	6.7 ± 0.3	12.8 ± 0.7	8.8 ± 0.5	8.0 ± 0.5	13.6 ± 2.5
-	Cohort Differences	High vs. Full: *t*=11.6, *p*<0.001	Full vs. Fair: *t*=0.7, *p*=0.480	High vs. Fair: *t*=10.8, *p*<0.001	Mild vs. Mod.: *t*=5.2, *p*<0.001	Mod. vs. Severe: *t*=5.6, *p*<0.001	Mild vs. Severe: *t*=0.2, *p*=0.810	Focused vs. Compr.: *t*=9.3, *p*<0.001	Focused vs. Compr.: *t*=9.3, *p*<0.001	2-5 vs. 6-12: *t*=1.1, *p*=0.269	6-12 vs. 13-18: *t*=3.5, *p*<0.001	2-5 vs. 13-18: *t*=3.1, *p*=0.003
Overall	ΔMean ± SEM	8.5 ± 0.1	15.8 ± 0.2	15.2 ± 0.2	11.4 ± 0.2	10.3 ± 0.1	14.1 ± 0.1	8.4 ± 0.1	18.5 ± 0.1	12.2 ± 0.1	11.5 ± 0.1	21.7 ± 0.8
-	Cohort Differences	High vs. Full: *t*=34.0, *p*<0.001	Full vs. Fair: *t*=2.1, *p*=0.036	High vs. Fair: *t*=31.8, *p*<0.001	Mild vs. Mod.: *t*=5.4, *p*<0.001	Mod. vs. Severe: *t*=26.8, *p*<0.001	Mild vs. Severe: *t*=13.3, *p*<0.001	Focused vs. Compr.: *t*=65.2, *p*<0.001	Focused vs. Compr.: *t*=65.2, *p*<0.001	2-5 vs. 6-12: *t*=4.8, *p*<0.001	6-12 vs. 13-18: *t*=25.6, *p*<0.001	2-5 vs. 13-18: *t*=22.8, *p*<0.001

All utilization cohorts showed overall improvement in skill acquisition success rates over the course of treatment; however, the high utilization cohort showed the smallest net improvement (8.5%) when compared with the full (15.8%) and fair (15.2%) utilization cohorts (Table 4). There was no consistent relationship between utilization and net improvement across the four focus areas. The greatest gains in communication were achieved by the full and fair utilization cohorts (14.7% and 14.3%, respectively). The greatest gain in emotional regulation was achieved by the fair utilization cohort (23.6%). The highest gain in terms of ΔMean success rate for any of the utilization cohorts was achieved by the full utilization cohort in executive functioning (27.7%). Finally, the greatest gains in social skills were achieved by the full and fair utilization cohorts (14.5% and 15.3%, respectively).

All utilization cohorts achieved statistically significant increases in success rates over time both overall and for all focus areas, with the exception of emotional regulation for the full utilization cohort (Table 5). The correlation coefficients (r) for the increase in success rates over the assessment period ranged from 0.486 to 0.796.

**Table 5:** Summary table indicating the correlation between week number and each focus area (shown as the Pearson correlation coefficient, r) and the t-statistic and p-value indicating the significance of the likelihood of the data having a true linear relationship (calculated using a Wald Test with a t-distribution of the test statistic, with the null hypothesis that the slope of the relationship is zero). *p < 0.05 indicates significant likelihood of data having a linear relationship with time.

-	-	COMM	-	-	ER	-	-	EF	-	-	SOC	-	-	Overall	-	-
-	-	r	t	*p*-value	r	t	*p*-value	r	t	*p*-value	r	t	*p*-value	r	t	*p*-value
Utilization cohort	High, n=91 (37.4%)	0.715	5.11	<0.001*	0.623	3.98	<0.001*	0.738	5.47	<0.001*	0.515	3.00	0.006*	0.796	6.57	<0.001*
-	Full, n=79 (32.5%)	0.669	4.51	<0.001*	-0.300	-1.57	0.129	0.486	2.78	0.010*	0.620	3.95	<0.001*	0.564	3.41	0.002*
-	Fair, n=73 (30.0%)	0.700	4.90	<0.001*	0.612	3.87	<0.001*	0.540	3.21	0.003*	0.657	4.36	<0.001*	0.656	4.35	<0.001*
ASD severity level cohort (DSM-5)	Mild, n=39 (16.0%)	0.504	2.92	0.007*	0.172	0.87	0.391	0.649	4.26	<0.001*	0.498	2.87	0.008*	0.735	5.42	<0.001*
-	Mod., n=106 (43.6%)	0.560	3.38	0.002*	0.207	1.06	0.300	0.805	6.79	<0.001*	0.403	2.20	0.037*	0.571	3.48	0.002*
-	Severe, n=98 (40.3%)	0.776	6.16	<0.001*	0.663	4.42	<0.001*	0.488	2.80	0.010*	0.753	5.72	<0.001*	0.744	5.57	<0.001*
Treatment plan type cohort	Focused, n=158 (65.0%)	0.640	4.17	<0.001*	-0.190	-0.97	0.342	0.570	3.47	0.002*	0.365	1.96	0.061	0.522	3.06	0.005*
-	Compr., n=85 (35.0%)	0.740	5.50	<0.001*	0.760	5.84	<0.001*	0.800	6.67	<0.001*	0.764	5.92	<0.001*	0.824	7.27	<0.001*
Age at first treatment session (years) cohort	2-5, n=96 (39.5%)	0.646	4.23	<0.001*	0.553	3.32	0.003*	0.737	5.44	<0.001*	0.653	4.31	<0.001*	0.726	5.28	<0.001*
-	6-12, n=128 (52.7%)	0.853	8.16	<0.001*	0.316	1.67	0.109	0.651	4.29	<0.001*	0.373	2.01	0.056	0.705	4.98	<0.001*
-	13-18, n=19 (7.8%)	-0.394	-2.14	0.042*	0.022	0.11	0.913	0.806	6.82	<0.001*	0.551	3.30	0.003*	0.610	3.85	<0.001*

ASD severity

Patients were stratified into ASD severity cohorts, namely, mild (n = 39, 16.0%), moderate (n = 106, 43.6%), and severe (n = 98, 40.3%), to assess how the ASD severity level affects success rates over time. In the mild, moderate, and severe ASD cohorts, the mean patient age in years was 7.3, 7.6, and 6.2, respectively, and patients with severe ASD were significantly younger than those with moderate ASD (Table 2). A majority of patients in each severity cohort had focused treatment plans, and the prescribed weekly treatment dosage was different for each severity cohort, with mild ASD having the least (20.9 hours), moderate ASD an intermediate amount (23.7 hours), and severe ASD the most (26.2 hours). In addition, each severity cohort contained patients with each of the three types of utilization (high, full, fair); however, the mild ASD cohort had significantly higher utilization (93.3%) than the moderate or severe ASD cohorts (83.0% and 82.7%, respectively).

**Figure 4:**
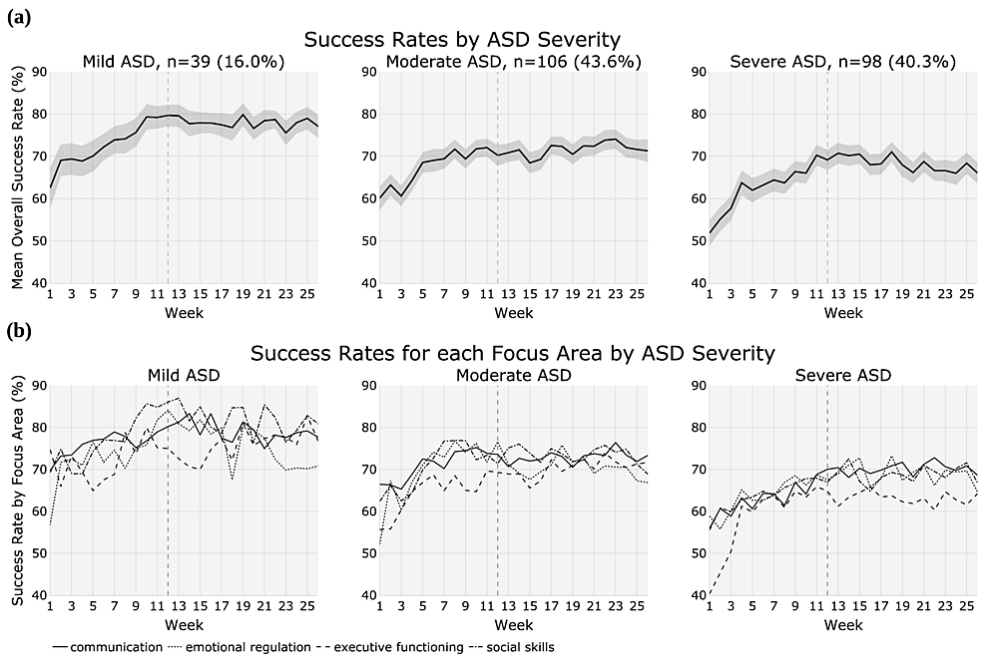
ASD severity level cohorts displaying: (a) mean success rate in overall skill acquisition and (b) success rate in skill acquisition in specific focus areas. ASD = autism spectrum disorder

The mean success rates over time for each severity cohort are presented in Figure 4a and Figure 4b. Table 6 details how many patients in each severity cohort received treatment in a given week. The severe ASD cohort demonstrated higher success rate gains from baseline to subsequent measurement, calculated as the ΔMean success rate in skill acquisition, compared to the mild and moderate ASD cohorts (Table 4). At baseline, the mild ASD cohort had its lowest success rate in emotional regulation and its highest success rate in executive functioning. The moderate ASD cohort similarly had its lowest baseline success rate in emotional regulation, but it showed its highest baseline success rate for communication. Within the severe ASD cohort, executive functioning showed the lowest and emotional regulation the highest baseline success rates. Executive functioning intermittently lagged behind other focus areas in terms of success rate throughout the treatment period for all severity cohorts. Although the moderate ASD cohort had the smallest overall increase in success rates (ΔMean), this cohort showed the most consistency in gains across all focus areas.

**Table 6:** Number of patients contributing to the reported data on a weekly basis for the ASD severity cohorts. ASD = autism spectrum disorder

Mild ASD, n=39 (16.0%)	-	-	-	-	-	-	-	-	-	-	-	-	-	-	-	-	-	-	-	-	-	-	-	-	-	-
Week number	1	2	3	4	5	6	7	8	9	10	11	12	13	14	15	16	17	18	19	20	21	22	23	24	25	26
Patients remaining	39	39	39	39	39	39	39	39	39	39	39	39	38	36	34	34	34	32	31	30	30	30	29	29	28	28
Patients on this week	39	38	38	36	37	37	38	38	38	39	38	38	37	35	33	34	34	31	31	30	30	30	27	27	26	28
Patients off this week	0	1	1	3	2	2	1	1	1	0	1	1	1	1	1	0	0	1	0	0	0	0	2	2	2	0
% of Patients off	0.0	2.6	2.6	7.7	5.1	5.1	2.6	2.6	2.6	0.0	2.6	2.6	2.6	2.8	2.9	0.0	0.0	3.1	0.0	0.0	0.0	0.0	6.9	6.9	7.1	0.0
Moderate ASD, n=106 (43.6%)	-	-	-	-	-	-	-	-	-	-	-	-	-	-	-	-	-	-	-	-	-	-	-	-	-	-
Week number	1	2	3	4	5	6	7	8	9	10	11	12	13	14	15	16	17	18	19	20	21	22	23	24	25	26
Patients remaining	106	106	106	106	106	106	106	106	106	106	106	106	103	98	94	93	91	84	79	77	75	73	67	67	63	62
Patients on this week	106	97	98	101	93	91	94	97	96	98	97	96	95	91	88	85	88	80	73	71	71	68	63	65	59	58
Patients off this week	0	9	8	5	13	15	12	9	10	8	9	10	8	7	6	8	3	4	6	6	4	5	4	2	4	4
% of Patients off	0.0	8.5	7.5	4.7	12.3	14.2	11.3	8.5	9.4	7.5	8.5	9.4	7.8	7.1	6.4	8.6	3.3	4.8	7.6	7.8	5.3	6.8	6.0	3.0	6.3	6.5
Severe ASD, n=98 (40.3%)	-	-	-	-	-	-	-	-	-	-	-	-	-	-	-	-	-	-	-	-	-	-	-	-	-	-
Week number	1	2	3	4	5	6	7	8	9	10	11	12	13	14	15	16	17	18	19	20	21	22	23	24	25	26
Patients remaining	98	98	98	98	98	98	98	98	98	98	98	98	96	93	93	91	91	82	79	76	76	75	74	72	70	63
Patients on this week	98	94	89	91	91	93	90	90	90	90	88	88	89	86	86	84	81	75	70	71	70	69	65	67	62	53
Patients off this week	0	4	9	7	7	5	8	8	8	8	10	10	7	7	7	7	10	7	9	5	6	6	9	5	8	10
% of Patients off	0.0	4.1	9.2	7.1	7.1	5.1	8.2	8.2	8.2	8.2	10.2	10.2	7.3	7.5	7.5	7.7	11.0	8.5	11.4	6.6	7.9	8.0	12.2	6.9	11.4	15.9

All severity cohorts showed improvement in skill acquisition success rates over the course of treatment; however, the severe ASD cohort showed the highest net improvement (14.1%), followed by the mild (11.4%) and moderate (10.3%) ASD cohorts (Table 4). The severe ASD cohort displayed marked improvements in communication (12.7%) and executive functioning (23.9%), with both focus areas showing a significant and consistent relationship between severity and gains (communication: moderate 6.9% and mild 7.9%; executive functioning: moderate 16.0% and mild 2.1%). Conversely, gains in emotional regulation were the highest in the mild and moderate ASD cohorts (14.0% and 14.7%, respectively) and lowest in the severe ASD cohort (5.8%). Finally, the greatest gains in social skills were achieved by the mild and severe ASD cohorts (11.6% and 11.3%, respectively), and these gains were significantly lower for the moderate ASD cohort (6.5%).

A significant positive correlation of increased success rate in skill acquisition over time was present for all three severity cohorts both overall and in communication, executive functioning, and social skills (Table 5). Emotional regulation showed a significant correlation only for the severe ASD cohort. Correlation coefficients (r) for the significant relationships varied, ranging from 0.403 to 0.805.

Treatment plan types

For the analysis by treatment plan type, patients were stratified based on having a focused (≤25 hours/ week; n = 158, 65.0%) or comprehensive (>25-40 hours/week; n = 85, 35.0%) treatment plan, to assess how this affects success rates over time. Patients with comprehensive treatment plans (mean age = 5.6 years) were significantly (t = 4.7, p < 0.001) younger than those with focused plans (mean age = 7.8 years) (Table 2). There was no significant difference in utilization between the treatment plan type cohorts. In addition, although each plan type cohort contained patients with each of the three severity levels (mild, moderate, and severe), a majority of patients in the comprehensive plan cohort had severe ASD (n = 44, 51.8%).

**Figure 5:**
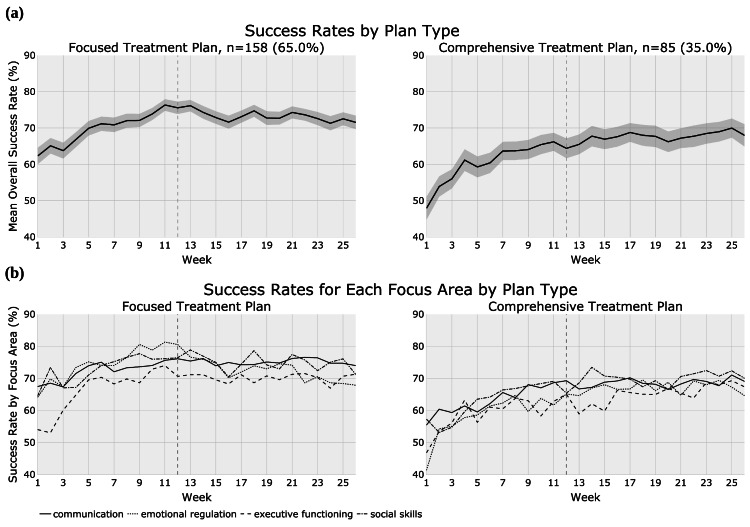
Treatment plan type cohorts displaying: (a) mean success rate in overall skill acquisition and (b) success rate in skill acquisition in specific focus areas.

The mean success rates over the study period for each treatment plan cohort are shown in Figure 5a and Figure 5b, and the number of patients contributing to the reported data on a weekly basis is shown in Table 7. Patients with comprehensive treatment plans consistently had lower success rates both at baseline and throughout the investigation period when compared with patients having focused treatment plans. Gains in executive functioning lagged behind other focus areas until week 21 for the focused cohort and intermittently for the comprehensive cohort. Patients in the comprehensive cohort achieved more than twice the overall ΔMean success rate compared to the focused cohort (18.5% vs. 8.4%, respectively; *t* = 65.2, *p *< 0.001; Table 4). The comprehensive cohort displayed a significantly greater ΔMean success rate in all focus areas when compared to the focused cohort: 13.6% versus 6.5%, respectively, in communication; 23.3% versus 3.7%, respectively, in emotional regulation; 20.6% versus 17.3%, respectively, in executive functioning; and 12.8% versus 6.7%, respectively, in social skills (*t* = 3.7-20.7 and *p *< 0.001 for all comparisons).

**Table 7:** Number of patients contributing to the reported data on a weekly basis by plan type cohorts.

Focused Plan, n=158 (65.0%)	-	-	-	-	-	-	-	-	-	-	-	-	-	-	-	-	-	-	-	-	-	-	-	-	-	-
Week number	1	2	3	4	5	6	7	8	9	10	11	12	13	14	15	16	17	18	19	20	21	22	23	24	25	26
Patients remaining	158	158	158	158	158	158	158	158	158	158	158	158	149	146	142	141	140	127	123	120	119	118	113	112	108	104
Patients on this week	158	145	144	148	144	139	142	141	144	144	140	139	139	137	135	130	130	119	116	114	113	108	104	106	97	97
Patients off this week	0	13	14	10	14	19	16	17	14	14	18	19	10	9	7	11	10	8	7	6	6	10	9	6	11	7
% of Patients off	0.0	8.2	8.9	6.3	8.9	12.0	10.1	10.8	8.9	8.9	11.4	12.0	6.7	6.2	4.9	7.8	7.1	6.3	5.7	5.0	5.0	8.5	8.0	5.4	10.2	6.7
Comprehensive Plan, n=85 (35.0%)	-	-	-	-	-	-	-	-	-	-	-	-	-	-	-	-	-	-	-	-	-	-	-	-	-	-
Week number	1	2	3	4	5	6	7	8	9	10	11	12	13	14	15	16	17	18	19	20	21	22	23	24	25	26
Patients remaining	85	85	85	85	85	85	85	85	85	85	85	85	82	77	76	75	74	65	62	61	60	57	55	52	51	48
Patients on this week	85	84	81	80	77	82	80	84	80	83	83	83	76	75	69	71	71	61	54	56	56	56	49	49	48	41
Patients off this week	0	1	4	5	8	3	5	1	5	2	2	2	6	2	7	4	3	4	8	5	4	1	6	3	3	7
% of Patients off	0.0	1.2	4.7	5.9	9.4	3.5	5.9	1.2	5.9	2.4	2.4	2.4	7.3	2.6	9.2	5.3	4.1	6.2	12.9	8.2	6.7	1.8	10.9	5.8	5.9	14.6

A significant positive correlation of increased success rate in skill acquisition over time was present for the comprehensive cohort both overall and in each of the four focus areas (*p* < 0.001 for all comparisons, *r *= 0.740-0.824, Table 5). The focused cohort only showed a significant correlation in overall success rates and in the communication (*r* = 0.640, *p *< 0.001) and executive functioning (*r* = 0.570, *p* = 0.002) focus areas.

## Discussion

This retrospective study evaluated patient outcomes from Forta’s pBT model, wherein parents deliver ABA treatment to their children under the ongoing supervision of BCBAs, longitudinally over six months of treatment. In previous work, we examined clinical outcomes in a relatively small patient cohort (n = 36) [34]. In this study, we utilized a larger patient cohort (n = 243), which allowed for a more robust assessment of the effectiveness of the pBT model, both overall and in the context of specific patient characteristics. In our primary hypothesis, we sought to examine if patients with severe ASD (per DSM-5) would achieve greater improvements over the course of the study period. We utilized pBT treatment documentation within our app to investigate success rates in skill acquisition in both overall and across multiple treatment focus areas. Consistent with our previous results, the severe ASD cohort showed significantly larger gains overall (ΔMean = 14.1%) than both the mild (ΔMean = 11.4%) and moderate (ΔMean = 10.3%) ASD cohorts. Our secondary hypothesis, that comprehensive treatment plans would result in greater skill acquisition gains than focused treatment plans, was substantiated by the robust and consistent gains attained by patients with comprehensive treatment plans across all focus areas. The results highlight sustained improvements from the pBT model overall. As exemplified in executive functioning for the full utilization cohort, patients initiating treatment with lower baseline skills had particularly dramatic gains. Thus, Forta’s novel pBT model enables significant improvements in patient outcomes in a clinical setting, as measured by skill acquisition across multiple focus areas.

Sustainable, real-world ABA treatment models require validation so that they may be broadly implemented to overcome barriers hindering treatment access, which include cost, provider shortages, and treatment delays. Families seeking ABA treatment for their children on the autism spectrum often face financial and logistical struggles related to obtaining ABA [28,51,52]. Many families also experience extended wait times to access ABA providers due to workforce shortages and growing demand for ABA due to increasing ASD prevalence [24,28]. The Forta pBT model eliminates many of the complexities associated with accessing treatment. Parents are empowered to provide direct, in-home ABA treatment to manage the core challenges of ASD, while benefiting from the rigor of professional oversight. By contrast, the skills gained during ABA in a clinical or research-only setting may not be as easily generalizable or reproducible outside of those environments [53]. As shown in previous work, patient progress toward skill acquisition in the Forta pBT model is equivalent to or better than in traditional treatment frameworks, establishing the pBT model as a functional and reliable method of ABA delivery [34]. The pBT model benefits from the development and validation of advanced technological tools to streamline data-driven treatment delivery [43,54]. This results in highly individualized ABA approaches to address each patient’s unique needs, which is imperative due to the heterogeneous nature of ASD [55]. Families with a child on the autism spectrum experience high levels of parental stress, social isolation, mental health disorders, and sibling struggles, many of which can also have adverse consequences for patient outcomes [51]. Providing families with the tools afforded within ABA to address the unique challenges of ASD can help mitigate many of these issues [56-59]. Altogether, the Forta pBT treatment model delivers accessible, high-quality ABA in addition to providing robust operational and clinical support for patients and their families [34,45].

Consistent with our previous research, the utilization of prescribed ABA treatment in our pBT model is extremely high (mean = 84.6%) [34]. Typically, 80% utilization of prescribed hours is considered a "full" dose of ABA treatment [42]. Although utilization is under-reported in the literature, our model appears to far exceed utilization typically attained in clinical settings; for example, Choi et al. (2022) found that only 28% of pediatric patients undergoing ABA treatment within a large health system received a full dosage of prescribed treatment [60]. Croen et al. (2017) reported that only 15% of pediatric patients referred for community-based ABA received the full prescribed hours [42,60]. In comparison, our treatment model shows that over 70% of Forta patients received a full dosage of prescribed ABA treatment. These results suggest that parents providing ABA within the pBT model are highly engaged and motivated to consistently implement treatment, which may result from overcoming many of the barriers to treatment accessibility, such as logistical issues, inadequately individualized treatment management, and insufficient availability of ABA services [33,52]. The stability of mean treatment utilization over the study period indicates this delivery method of ABA treatment via pBTs is sustainable. The high utilization cohort showed both the least variability in longitudinal success rates and the highest baseline communication success rates. This may indicate that communication (e.g., language skills) can influence treatment effectiveness; higher communication abilities have previously been reported to correlate with improved adaptive behavior and intellectual functioning [16,61].

In contrast to the other utilization cohorts, progress in executive functioning for the high utilization cohort was similar to other focus areas; the full and fair utilization cohorts showed less progress in executive functioning in comparison to other focus areas. This suggests that executive functioning may be the area with the most room to benefit from higher utilization of prescribed treatment. There were age-related differences in treatment outcomes within the utilization cohorts, with the youngest patients (two to five years) in the fair utilization cohort showing lower mean gains overall compared to older patients (six to 12 years and 13-18 years). As some previous studies have also suggested, this may indicate that very young children on the autism spectrum are more sensitive to differences in treatment dosage and intensity and can benefit from a higher dosage of ABA [16,62]. Early and intensive (i.e., higher dosage) ABA treatment, when children are still developing language skills, can have a greater impact on outcomes for children on the autism spectrum [15,63]. Consistent with this concept, teenage patients (13-18 years) had relatively limited improvement in communication skills but higher improvements in executive functioning, emotional regulation, and social skills (Table 4). While these differences between the age cohorts were statistically significant, the limited sample size in the teenage cohort of only 19 individuals may reduce the generalizability of these results. Age-related differences in treatment response may be influenced by factors, such as developmental level or changes in language skills over time. For instance, as children on the autism spectrum get older, they may become more focused on social relationships and less interested in developing communication skills [64]. In addition, teenage patients may already have developed some adaptive behaviors, making it more challenging to make substantial gains in communication [65].

ASD severity had a significant impact on treatment outcomes. A significant correlation of increased net gains in skill acquisition over time, overall and for three of four focus areas across all ASD severity cohorts (Table 5), demonstrated the broad success of the pBT model. Patients with mild and moderate ASD made consistent gains across all focus areas with the exception of emotional regulation skills. In addition, patients in the severe ASD cohort achieved statistically significant improvements in emotional regulation, in contrast to the literature indicating that adaptive behavior, a component of emotional regulation, is easier to attain for individuals with mild ASD [66]. The moderate and severe ASD cohorts showed variable gains in individual focus areas; however, in the mild and moderate ASD cohorts, success rates stabilized at approximately 80% and 70%, respectively. This may reflect the fact that patients transitioned into more challenging or longer-term goals after rapidly attaining the skills of introductory goals during treatment initiation. Patients in the severe ASD cohort demonstrated large improvements in executive functioning and communication, showing the highest net improvement of any of the ASD severity cohorts. Although the severe ASD cohort had the highest amount of prescribed ABA hours, they did not reach the same level of skill acquisition success rates from baseline through the conclusion of the study in emotional regulation and social skills. This may reflect the need to provide individuals with severe ASD with longer duration ABA treatment to achieve optimal clinical gains, which are typically made only after 24 months of ABA treatment [60]. Despite their slower pace of improvement across focus areas, patients with mild ASD were able to attain mastery (typically 80-100% success rate), in most areas due in part to their high baseline at treatment initiation [46].

The type of treatment plan assigned to patients (focused vs. comprehensive) also had a significant impact on treatment outcomes. The comprehensive cohort (>25-40 hours/ week) had a significant correlation of increased success rates in skill acquisition over the study period both overall and in all focus areas. This aligns with the literature suggesting that ABA treatment intensity is a predictor of patient outcomes, exhibiting a dose-response relationship across multiple domains [15]. This may account for the higher overall net gains achieved by the comprehensive cohort in comparison to the focused cohort. A meta-analysis by Eckes et al. (2023) reported that comprehensive ABA achieved greater improvement in adaptive behavior than either non-comprehensive ABA or non-ABA treatments [16]. Patients with comprehensive treatment plans showed larger gains in communication, social, and emotional regulation skills compared to those with focused treatment plans, despite having lower overall goal success rates throughout the duration of treatment. These differences may be due to the comprehensive cohort having a majority of patients with severe ASD (n = 44, 51.8%). This aligns with the principle that comprehensive plans are better suited for individuals with more severe symptoms or complex needs [16]. Furthermore, given that across all severity levels, the severe ASD cohort demonstrated the highest net gains overall, and in communication and executive functioning, it is likely that the success observed for the comprehensive cohort overall and in these two focus areas is in part owed to having a majority of patients with severe ASD.

Study limitations

Our study has demonstrated the value of Forta’s pBT ABA model in improving patient outcomes within multiple skill acquisition focus areas and across different levels of ASD severity, treatment dosage, and utilization and fills a gap in research related to a parent-led model of ABA in practice, as opposed to strictly in a research setting. However, there are limitations to the generalizability of these findings. First, our study relies on a convenience sample, which may not be representative of the broader population; therefore, different outcomes may be found using other patient populations. Early and adequate levels of ABA treatment, particularly for younger children, may influence long-term progress across focus areas [67]. Within our pBT model, robust operational support is leveraged in order to onboard families as quickly as possible while maintaining compliance for ABA training and insurance. In the few studies that examine treatment delay, results suggest that later treatment initiation can negatively impact treatment outcomes [68,69]. While this study examines a variety of demographic and treatment-specific factors to determine their impact on treatment progress, ASD is a highly complex, heterogeneous diagnosis, where outcomes are influenced at the society, family, and individual levels [7,37,70]. Therefore, while these results shed light on variables that can influence treatment progress, this work is not comprehensive of all ASD aspects. The training required as part of the pBT model is extensive and therefore may not be reproducible in populations of parents where time to allocate to training and/or providing treatment is limited. In addition, generalizability is unknown within subpopulations of individuals identifying as ethnic or racial minorities. However, it should be noted that from the pBTs that chose to identify their race or ethnicity, the majority were non-White (Table 1).

Future directions

Future work could expand on this study to gain a more in-depth understanding of progress throughout ABA treatment and the impact of a wider array of specific patient characteristics. Larger and more diverse cohorts, randomly selected to represent the heterogeneous population of children on the autism spectrum, could be utilized to assess the impact of treatment delays on clinical outcomes. To advance our understanding of the relationships between treatment outcomes and patient characteristics, we propose several areas for further investigation. Parent age at birth and baseline cognition, which have been shown to influence treatment outcomes, need further investigation [71,72]. As Forta’s patient population continues to grow and diversify, we will be uniquely positioned to examine ABA from a cultural perspective. This will be invaluable to fill gaps in research on culturally adapted ABA, which can improve patient outcomes for the targeted populations [73,74]. The Cultural Formulation Interview and the development of culturally adapted programming in the pBT model could be implemented to examine acceptability, parent confidence, and patient clinical outcomes [75]. This is essential for understanding the diverse needs of individuals across the autism spectrum and will help tailor treatment to better serve a broad population of individuals [76]. Lastly, future studies should investigate how treatment planning can be refined to help identify the most effective plans for specific subgroups based on diverse patient characteristics to help optimize treatment outcomes [16].

## Conclusions

The continued increase in ASD prevalence and the resulting demand for therapists qualified to provide treatment calls for innovative approaches for evidence-based treatment, such as ABA, which can mitigate the social and daily functioning struggles experienced by individuals on the autism spectrum. To that end, over a six-month period, we demonstrated the longitudinal effectiveness of a novel pBT model for ABA treatment delivery using the metric of success in skill acquisition. Our findings revealed that patients within the pBT model showed significant progress in skill acquisition both overall and within specific focus areas, regardless of cohort stratification. Patients with severe ASD demonstrated larger gains in overall skill acquisition than those with mild or moderate symptoms. In addition, patients with comprehensive treatment plans achieved greater gains toward their skill acquisition goals compared to those with focused treatment plans. This study highlights the importance and feasibility of parent-delivered ABA as an effective approach for addressing challenges in accessing appropriate treatment faced by families with a child on the autism spectrum. When parents are provided with training, tools, and ongoing support for the delivery of at-home ABA treatment, they are empowered to actively participate in their child’s development. We provide strong evidence that this innovative pBT model is an effective and sustainable solution for delivering ABA treatment. High utilization rates, parental involvement, and our data-driven treatment tools improve clinical outcomes for children on the autism spectrum and support families throughout the treatment process.
